# “Teaching old dogs new tricks” – POCUS Education for Senior Faculty

**DOI:** 10.24908/pocus.v8i1.16145

**Published:** 2023-04-26

**Authors:** Daniel Restrepo, Thomas F Heyne, Christine Schutzer, Renee Dversdal

**Affiliations:** 1 Department of Medicine, Massachusetts General Hospital Boston, MA USA; 2 Harvard Medical School Boston, MA; 3 Point-of-Care Ultrasound Program, Oregon Health & Science University Portland, OR; 4 Division of Hospital Medicine, Oregon Health & Science University Portland, OR

**Keywords:** Senior Faculty, Faculty Development, Graduate Medical Education, Curriculum development

## Abstract

Point of Care Ultrasound (POCUS) is a growing diagnostic modality across a variety of specialties and is increasingly being taught in undergraduate medical education. Uptake within internal medicine has been slow but is becoming more commonplace. Training of extant hospital medicine faculty, including senior members, in POCUS is an unmet need in graduate medical education with significant pedagogical and patient safety implications. With this in mind, we created a training program for the core teaching faculty at our academic internal medicine residency program. The experiential, hands-on curriculum explored the reasoning behind concepts and emphasized psychological safety for senior faculty learners and was successful and well-received. In our piece, we aim to explore the existing literature around training this unique population in POCUS and report on our single-center experience. We also provide a framework for how our program succeeded, collate tips derived from the expert ultrasound teachers and list pearls learned while teaching these experienced educators. Although this worthwhile effort requires planning and support, it was appreciated even by senior faculty.

## Introduction

Over the past 25 years, the practice of point of care ultrasound (POCUS) has vastly expanded and is now utilized across a variety of specialties ranging from emergency medicine to rheumatology. POCUS differs from consultative or comprehensive ultrasound imaging in that it is performed by the patient’s clinician at the bedside to answer a focused set of questions that aid in diagnosis 1. Though initially slow to uptake the skill compared to other specialties, internists have begun to utilize POCUS to care for both inpatients and outpatients 2. Simultaneously, incorporation of POCUS education in undergraduate medical education curricula has grown, with now almost 73% of medical schools including POCUS in their preclinical courses as well as clinical education 3. This poses a unique challenge within academic internal medicine, as many incoming first-year medical residents will arrive each year trained in skills that their teachers lack. Modern medical residents are eager to use POCUS to care for patients, however at least one study has shown significant amount of diagnostic inaccuracy in residents with cursory training 4. It is possible and indeed likelier every day, that a resident will start to make diagnostic and therapeutic decisions using POCUS. Thus, training of extant faculty in POCUS represents a growing need amongst internal medicine residency training programs nationwide, one that has important patient safety implications as well. In this perspective piece, we seek to denote the challenges in teaching senior learners a dynamic and multifaceted skill such as POCUS and to report on our experience in training such a group at one institution. 

## Challenges in teaching senior faculty

Mid-to-late career faculty, herein defined as faculty with at least 5 years of practice since completing training, represent a unique population from a pedagogical standpoint and one for which there is sparse educational guidance in the literature. The data available for POCUS education in this population within the internal and emergency medicine literature would suggest that these learners are able to learn POCUS at similar rates to trainees^5^ but may ultimately not incorporate it into their clinical practice 6. In many ways, they are the standard adult learner: self-motivated and ready to learn if the subject matter is relevant to their goals. Employing the principles of learning science is likely to be high-yield, and thus the curriculum should be experiential, problem-focused and presented in a way that both emphasizes the “why” and allows for discovery rather than emphasizing memorization 7. However, many individuals in this career stage are likely to be seasoned or expert clinicians who have not sat in the novice’s seat for some time and thus may feel vulnerable in the process of acquiring new skills. There must be an emphasis on creating psychological safety in the teaching of a new skill, especially if taught in a group setting amongst peers. Additionally, there are likely to be kinesthetic and spatial learning considerations with regards to POCUS image acquisition and interpretation. With initial training, the emphasis for mid-career or senior faculty should be on comfort over competence. Achieving competence in this complex clinical skill can be overwhelming and the goal at the end of an introductory course should be that individuals should be comfortable enough with the technology and interpretation to begin practicing on their own between scheduled trainings. Techniques such as task deconstruction for the specific transducer movements can go a long way to establishing a strong foundation in the fundamental skills of POCUS. Implementing measures such as task deconstruction for a kinesthetic skill such as POCUS is especially relevant to those who practice in a predominantly cognitive specialty like internal medicine, and for those who likely have not performed bedside procedures since their residency training. The opportunity for repeated, hands-on practice either on a POCUS model or a live volunteer with consistent, calm, and non-judgmental instruction is key. The goal as instructors is for the faculty to feel comfortable at the end of the introductory course and achieve competency at the end of longitudinal training. 

## The MGH Experience

Acknowledging the need for POCUS-trained faculty in our Internal Medicine Residency Program, we set out to begin training our Core Educator Faculty in POCUS during the fall of 2019. Our goal was to pursue a “train-the-trainer” approach wherein a cadre of dedicated POCUS teachers could more easily train the much larger population of residents in POCUS. The Core Educator Faculty (CEF) for the Massachusetts General Hospital Internal Medicine Residency Training Program is a 16-member group of hospitalist clinician-educators at varying career stages who are responsible for most of the residency’s clinical teaching on the inpatient medical services. All members of the CEF had completed residency training more than five years prior, and none had any formal education regarding diagnostic POCUS. Many in the group had been in practice for more than 20 years and had never held an ultrasound probe or interpreted ultrasound images before. 

We designed a so-called “bolus/drip” curriculum wherein the dissemination of content was accomplished in an intensive and hands-on single-day retreat – representing the “bolus.” Following this, a select group of 3-4 faculty “super-users” would engage in longitudinal competency acquisition over the subsequent months – the “drip.” Once these select faculty members completed the longitudinal training, a new set of “super-users” would be selected for intensive training. 

The “bolus” was taught by a group of 3 hospitalists with extensive POCUS education experience as well as an educational sonographer. This “bolus” training consisted of 6 total POCUS applications: heart, lung, inferior vena cava (IVC)/jugular venous pressure, abdominal fluid evaluation (Focused Assessment of Free Fluid – or FAFF) 8, renal/bladder and soft tissue POCUS. Both handheld and cart-based ultrasound machines were used for teaching purposes in order to expose learners to different device types. The day-long session was designed to employ didactic modules for content dissemination followed immediately by hands-on practice sessions on live human models. The learning content covered the anatomy for each POCUS application, scanning technique, the normal sonographic findings, and a brief overview of select pathologic states. This hybrid didactic/hands-on model was intentional and served to punctuate the large amount of didactic material being presented with breaks in which direct application of the content could be performed. We designed the session to have a low faculty-to-learner ratio with only three or four learners per station with one faculty member. 

The “drip” was largely comprised of scheduled and ad hoc scanning sessions taught by a designated ultrasound-fellowship-trained hospitalist. Following the “bolus”, CEF members interested in more intensive training were asked to write a short paragraph to be selected as one of three “super-users” and were considered based on level of interest, bandwidth and overall clinical footprint on the teaching services. These super-users met with the trainer to scan live inpatients who either volunteered for an educational scan or for whom a scan was clinically warranted. These scanning sessions occurred up to three times per month and lasted around two hours. In addition, all faculty were offered roughly monthly virtual sessions for image review, as a content refresher. To complete their training, each “super user” was required to complete a total of 150 scans (25 scan per application), including both supervised and unsupervised scans. The latter were reviewed by the trainer, and asynchronous feedback was given. After finishing their scanning portfolio, the “super user” was required to complete an objective structured clinical examination (OSCE) with the trainer. These minimum standards were derived from the American College of Emergency Physicians’ training guidelines^9^ for POCUS and were finalized in collaboration with our Department of Radiology, Department of Emergency Medicine, and the MGH Credentialing Office. Upon completion of training and credentialing, the faculty members were given hospital privileges to perform diagnostic POCUS and bill on their scans. Subsequent clinical scans were reviewed for quality assurance by the trainer. In general, most super-users took 12-18 months to complete their training. Notably, this method inherently creates a large gap in POCUS exposure for faculty members not selected as super-users while the initial cadre are trained, during which time content knowledge and skills are likely to dissipate. To remediate this, all faculty are encouraged to attend the virtual refresher sessions noted above and the subsequent batch of super-users undergo a quick refresher upon selection.

## Reflections, Tips and Challenges

Our experience in teaching a cadre of mid-career to senior faculty was an extraordinarily positive one. Upon beginning the intensive “bolus”, it became clear that the faculty were simultaneously ecstatic and apprehensive about learning a new skill. Our plan at the outset was to maximize the benefit of frequent breakouts and time with hands on a probe. Often, we found most benefit in leveraging the physical examination knowledge that the faculty possessed – correlations of the external examination of the jugular venous pulse to that of the sonographic internal jugular assessment or the IVC were the subject of joy and intense discussion. A feature that was surprising, but not unexpected, was the degree with which these experienced teachers asked “why” when presented with new material. It was noted several times during the training program that these educators would ask their instructors to delve into details that more junior learners such as residents seldom investigate - whether due to comfort with interrupting a teacher due to inherent seniority or because their teaching had for so long consisted of deconstructing mechanisms or processes is unclear. Lastly, we found great success with spaced learning and weaving prior material into the curriculum – for example, using callbacks to similar anatomy shared in different scanning applications such as that seen when imaging the right flank during the sonographic assessment of free abdominal fluid or when searching for a pleural effusion. The training program was very well-received amongst the Core Educator Faculty and they showed an infectious enthusiasm for acquiring new skills, even amongst those not selected as a super-user later. Multiple faculty members, some decades into their careers, remarked on the joys of learning a new skill, one which allowed them to see into the human body at the bedside like they never had before. These faculty members frequently spontaneously voiced these sentiments as having the potential to mitigate burnout. Additionally, through our continuing educational review sessions, non-super users have acquired skills in image interpretation despite not recalling how to acquire them – which is in keeping with what others have found in training similar populations using asynchronous cognitive-focused curricula 10. The faculty who completed their super-user training continues to scan actively and use POCUS in their clinical decision making on a near daily basis and are actively engaged in our image review sessions. We have currently completed training for five faculty members and are actively training four additional ones. Many of the strategies utilized and phenomena witnessed share a great deal in common with principles of learning science and are universally applicable in most spheres of medical education. We have outlined the tips and tricks we felt most useful in Table 1.

**Table 1:** Tips and tricks in the teaching of diagnostic point of care ultrasound (POCUS) for senior faculty learners

**Tip**	**Description**
Develop a hook	As with most learners, engagement is made much more likely if senior faculty have vested interest in learning the material. Utilizing elements of lifelong learning, patient safety and the rising numbers of POCUS-savvy house officers are powerful motivators.
Use callbacks	Reference prior images, scanning zones or probe handling techniques through spaced learning and delayed recall. If possible, attempt to have the learners recall similarities or homologous concepts from other scanned areas. This will allow learners to revisit information and create cognitive connections that strengthen learning.
Teach the “why”	As opposed to junior learners, senior faculty are more likely to expect their instructors to know why something is the way it is. This may result in longer time to teach concepts and require instructors to have a strong grasp of abstract topics (e.g. mirror image artifact) though it may possibly lead to stronger associations and better learning.
Leverage existing knowledge	Whenever possible compare the new skill (POCUS) to skills which the senior learner has already mastered such as aspects of the physical exam with direct relevance such as the jugular venous pressure, S3s, fluid waves or egophony and sonographic findings of JVD, IVC plethora, left ventricular dysfunction and ascites.
Maximize hands-on practice	Senior learners are likely to grasp concepts quickly however may have the most issue with kinesthetic skills and learning of a procedural task. Additionally, the inherent spatial considerations of scanning zones and POCUS images may be difficult for some senior learners. Spend the most time possible during introductory stages with hands on a probe.

## Conclusion

The popularization of POCUS amongst hospitalists, coupled with the rising rates of ultrasound education at the undergraduate medical education level will likely lead to a large need in training of sizeable numbers of faculty to keep up with the new skills of their learners. We have found that training academic hospitalists in POCUS is feasible and well-received even by senior faculty, although this worthwhile effort requires planning and support. 

## Conflicts of Interest

RKD served on the board of governors of the American Institute of Ultrasound in Medicine until 7/2020. She has been the Chief Medical Officer at Vave Health since 7/2020, however the bolus training and her involvement in this research had concluded by then. CMS received consulting fees from the Global Ultrasound Institute. DR and TFH have no conflicts of interest to declare.

## References

[R178650627470909] Soni N J, Lucas B P (2015). Diagnostic point-of-care ultrasound for hospitalists. J Hosp Med.

[R178650627470913] Bhagra A, Tierney D M, Sekiguchi H, Soni N J (2016). Point-of-Care Ultrasonography for Primary Care Physicians and General Internists. Mayo Clin Proc.

[R178650627470907] Nicholas E, Ly A A, Prince A M, Klawitter P F, Gaskin K, Prince L A (2021). The Current Status of Ultrasound Education in United States Medical Schools. J Ultrasound Med.

[R178650627470911] Ojeda J C, Colbert J A, Lin X, McMahon G T, Doubilet P M, Benson C B, Wu J, Katz J T, Yialamas M A (2015). Pocket-sized ultrasound as an aid to physical diagnosis for internal medicine residents: a randomized trial. J Gen Intern Med.

[R178650627470914] Yamada T, Minami T, Soni N J (2018). Skills acquisition for novice learners after a point-of-care ultrasound course: does clinical rank matter?. BMC Med Educ.

[R178650627470906] Smalley C, Simon E, Muir M, Delgado F, Fertel B (2021). Point-of-Care Ultrasound Training and Credentialing for mid-late Career Emergency Physicians: Is it worth it? Credentialing Late Career Emergency Physicians. POCUS Journal.

[R178650627470910] Knowles M S (1984). Applying Modern Principles of Adult Education.

[R178650627470915] Maitra S, Jarman S D, Halford S W (2008). When FAST is a FAFF: is FAST Scanning Useful in Non-Trauma Patients?. Ultrasound.

[R178650627470908] (2017). Ultrasound Guidelines: Emergency, Point-of-Care and Clinical Ultrasound Guidelines in Medicine. Ann Emerg Med.

[R178650627470912] Anstey J, Jensen T, Lalani F, Conner S M (2022). Teaching the Teachers: A Flexible, Cognitive-Focused Curriculum in Point-of-Care Ultrasound Education for Hospital Medicine Faculty. J Ultrasound Med.

